# Extensive analysis of D-J-C arrangements allows the identification of different mechanisms enhancing the diversity in sheep T cell receptor β-chain repertoire

**DOI:** 10.1186/1471-2164-11-3

**Published:** 2010-01-04

**Authors:** Silvia Di Tommaso, Rachele Antonacci, Salvatrice Ciccarese, Serafina Massari

**Affiliations:** 1 Dipartimento di Scienze e Tecnologie Biologiche ed Ambientali, Universita' del Salento, Lecce, Italy; 2 Dipartimento di Genetica e Microbiologia, Universita' degli Studi di Bari, Bari, Italy

## Abstract

**Background:**

In most species of mammals, the *TRB *locus has the common feature of a library of *TRBV *genes positioned at the 5'- end of two in tandem aligned D-J-C gene clusters, each composed of a single *TRBD *gene, 6-7 *TRBJ *genes and one *TRBC *gene. An enhancer located at the 3'end of the last *TRBC *and a well-defined promoter situated at the 5'end of the *TRBD *gene and/or a undefined promoter situated at the 5'end of the *TRBD2 *are sufficient to generate the full recombinase accessibility at the locus. In ruminant species, the 3'end of the *TRB *locus is characterized by the presence of three D-J-C clusters, each constituted by a single *TRBD*, 5-7 *TRBJ *and one *TRBC *genes with the center cluster showing a structure combined with the clusters upstream and downstream, suggesting that a unequal crossover occurred in the duplication. An enhancer downstream the last *TRBC*, and a promoter at the 5'-end of each *TRBD *gene are also present.

**Results:**

In this paper we focused our attention on the analysis of a large number of sheep TR β-chain transcripts derived from four different lymphoid tissues of three diverse sheep breed animals to certify the use and frequency of the three gene clusters in the β-chain repertoire. As the sheep *TRB *locus genomic organization is known, the exact interpretation of the V-D-J rearrangements was fully determined. Our results clearly demonstrate that sheep β-chain constitutes a level of variability that is substantially larger than that described in other mammalian species. This is due not only to the increase of the number of D and J genes available to the somatic recombination, but also to the presence of the trans-rearrangement process. Moreover, the functional complexity of β-chain repertoire is resolved by other mechanisms such as alternative cis- and trans-splicing and recombinational diversification that seems to affect the variety of the constant region.

**Conclusion:**

All together our data demonstrate that a disparate set of molecular mechanisms operate to perform a diversified repertoire in the sheep β-chain and this could confer some special biological properties to the corresponding αβ T cells in the ruminant lineage.

## Background

Mature T lymphocytes must express heterodimeric α and β or γ and δ chain T cell receptors (TRs) on its surface in order to provide protection from pathogens. The diversity of the TR repertoire derives in large part from the random somatic rearrangements of Variable (V), Diversity (D) and Joining (J) genes in the case of δ and β chain, and Variable (V) and Joining (J) genes in the case of γ, and α chain encoding the variable portion of these molecules during the T-cell differentiation.

The V(D)J process requires the binding of the lymphocyte-specific recombination activating gene 1 and 2 (RAG1/2) protein complex to recombination signal sequences (RSs) flanking the rearranging sides of the individual V, D and J genes [1]. Upon binding, the RAG1/2 recombinases introduce a nick at the border between the RS heptamer and the adjacent coding sequence. The DNA repair factors of the nonhomologous end-joining (NHEJ) machinery join the nicked genes [2]. The RSs consist of conserved heptamer and nonamer sequences, separated by a spacer of 12 or 23 bp of relatively non-conserved DNA. Efficient recombination involves pairs of genes flanked by dissimilar 12- and 23RSs (the 12/23 rule) [3]. However, at the locus encoding for the β-chain (*TRB*), despite the 12/23 compatibility, the *TRBD *12RSs, but not the *TRBJ *12RSs efficiently target Vβ 23RSs. This phenomenon termed "beyond 12/23 rule" [4], preserving the *TRBD *gene utilization, ensures an ordered V(D)J recombination at the *TRB *locus with the *TRBD*-to-*TRBJ *joining which occurs before the *TRBV*-to-*TRBD *gene assembly.

Diversity at the recombination level is further enhanced by other processes that include the exonuclease digestion (trimming) of 3'-V, 5'- and 3'-D, and 5'-J genes, the imprecise joining of nicked genes, and the addition of non germline nucleotides (N nucleotides) at the V-J, V-D and D-J junctions. For this reason the product of the V(D)J joining, corresponding to the CDR3 region in the chain, is markedly polymorphic and is dominant in the recognition of peptide. After transcription, the V(D)J sequence is spliced to the constant (C) gene.

The resources available to generate the potential repertoires and to establish the regulation are described by the genomic organization of the TR loci. In most species of mammals, the *TRB *locus has the common feature of a library of *TRBV *genes positioned at the 5'- end of two in tandem aligned D-J-C gene clusters, each composed of a single *TRBD*, 6-7 *TRBJ *and one *TRBC *genes, followed by a single *TRBV *gene with an inverted transcriptional orientation located at the 3'-end. This genomic organization is reported well conserved from human [5], mouse [6,7], rat [8], chimpanzee [9], rhesus monkey [10], and horse [11]. A peculiar feature of the mammalian *TRB *locus is the presence of two very similar *TRBC *genes, since they differ by only a few residues in the coding region; conversely, they are different in their own 3'-UTR regions.

In the artiodactyls lineage, i.e., in sheep [12] as well as in cattle [13] and in pig [14], a duplication event within the 3'-end of the *TRB *locus has led to the generation of a third D-J-C cluster. The presence of an additional cluster produces an increase in the number of D and J genes available to partake in somatic recombination, but also expand the distance between the enhancer (Eβ) and the promoter (PDβ1) elements within the locus. Surprisingly also, in presence of three D-J-C clusters, both the nucleotide and protein sequences of all three *TRBC *genes are highly similar. Only four amino acid residues have undergone replacement in the *TRBC1 *gene with respect to the *TRBC2 *and *TRBC3 *genes, while the *TRBC3 *3'-UTR region is identical to that of *TRBC1 *gene [12]. The amino acid replacements were located, two in the N- terminus and one in the E β-strand and in the FG loop of well-defined regions of the extracellular domain of the TRBC molecule [15].

To know if the altered genomic architecture of the ruminant *TRB *locus can modify the mechanisms of recombination, we investigated on the β-chain repertoire in sheep. For this purpose we produced a collection of cDNAs derived from four different tissues belonging to four different adult animals of three diverse sheep breeds. As the genomic organization is known, the exact interpretation of the β chain transcripts was determined. The results of the analyses clearly demonstrate that sheep possess a repertoire of functional TRβ genes that is substantially larger than that described for other mammalian species, but also that other mechanisms as trans-rearrangement, intrallelic trans-splicing and DNA recombinational diversification involving the constant regions seem to shape the β-chain repertoire in a consistent way. However, the general paradigms of the mammalian *TRB *regulation seem to be preserved.

## Results

### Analysis of β-chain transcripts

**Figure 1:**
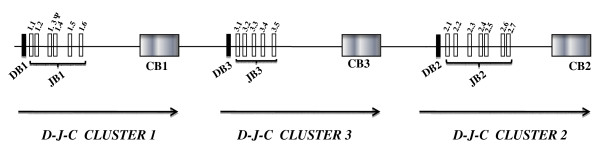
**Schematic representation of the genomic organization of the 3'-end of the sheep *TRB *locus from *TRBD1 *to *TRBC2 *genes (modified from fig. 1 by Antonacci et al**. [12]).

A previous study on cloning and sequencing of the sheep *TRB *locus revealed that the D-J-C region is organized in three independent clusters tandem aligned, with D-J-C cluster 3 additional with respect to the other mammalian *TRB *loci [12]. D-J-C cluster 1 contains one *TRBD*, six *TRBJ *and one *TRBC *gene. D-J-C cluster 3, located at 2.4 Kb downstream cluster 1, includes one *TRBD*, five *TRBJ *and one *TRBC *gene. Finally, D-J-C cluster 2 is positioned at 2.6 Kb downstream cluster 3 with one *TRBD*, seven *TRBJ *and one *TRBC *gene (fig. 1).

To evaluate the contribution of each gene cluster in the formation of the β-chain repertoire, a total of 72 clones containing rearranged V-D-J-C transcripts with a correct open reading frame were analyzed. All cDNA clones were registered in EMBL database with the Accession numbers from FM993913 to FM993984. 21 of these clones were derived from perinatal thymus (pSTMOS series) of a *Moscia Leccese *breed sheep, 15 from adult thymus (pSTA series) and 19 from spleen (pSMA series) of a *Gentile di Puglia *breed sheep, 17 from peripheral blood (pSSAR series) of a *Sarda Ionica *breed sheep. The clones were obtained by RT-PCR. The 5' primer was chosen on the YLCASS amino acid motif of the *TRBV *genes as members of the *TRBV *subgroups with this motif which seem to be the most frequently used [16] while the 3'-primer was designed on a conserved region of the three *TRBC *genes [12].

The deduced amino acid sequences of the V-D-J regions of all 72 cDNA clones are reported in the Table 1 together with the corresponding *TRBC *genes, according to the tissue of origin. Among the clones only one sequence is shared between blood (pSSAR25) and adult thymus (pSTA03). No tissue-specific expression of the genes was found. A total of 16 *TRBJ *genes were recovered within the different cDNAs. Thus, only one out of 17 functional *TRBJ *genes present in the genomic sequence was completely absent (*TRBJ2.6*). Besides, all TRBJ sequences match well with the corresponding genomic ones, and the high level of sequence similarity observed among the different animals is consistent with a close phylogeny of sheep breeds. The *TRBJ2 *cluster seems to be preferentially used (38/72 = 52.7%) and, although the numbers are too low to be statistically relevant, a slight increase in the use of *TRBJ2.3 *(14/38 = 36.8%) and *TRBJ2.7 *(10/38 = 26.3%) genes can be observed. Moreover, 20 clones retain a member of the *TRBJ3 *cluster, with the *TRBJ3.4 *gene (9/20 = 45%) more frequently used, while 14 clones retain the *TRBJ1 *gene set, without any preferential usage.

**Table 1:** Predicted amino acid sequences and length of the junctional diversity of the cDNAs. The classification of the *TRBD*, *TRBJ *and *TRBC *genes is indicated.

	CDR3 sequence							
						
CLONE	V	N(D)N	J	Ac.N	D segment	J segment	C segment	CDR3 length
pSTMos.01	ASSQ	A(GT)VI	SYEQYFGPGTKLTVV	FM993913	D1	J2.7	C2	14
pSTMos.02	ASSPT	NIAY	SYEQYFGPGTKLTVV	FM993914	--	J2.7	C2	14
pSTMos.03	ASSQ	SD(G)	EQYFGPGTKLTVV	FM993915	D3	**J2.7**	**C3**	10
pSTMos.04	ASSP	PP(GQ)V	NTEVFFGKGTRLTVV	FM993916	D1	J1.1	C1	14
pSTMos.05	ASSQG	R(D)L	SNNPLYFGGGTRLLVL	FM993917	D1	**J2.3**	**C3**	14
pSTMos.06	ASSQG	P	NTDPLYFGAGSKLTVL	FM993918	--	J2.2	C2	12
pSTMos.07	ASSR	VGTME(GQ)I	YNSOLQFGIGTRLTVT	FM993919	D1	J1.6	C1	18
pSTMos.08	ASSPT	E(GLWGG)R	YEQYFGPGTKLTVV	FM993920	D2	**J2.7**	**C3**	16
pSTMos.09	ASSQE	AR(LG)AR	YFGAGTRLSVL	FM993921	D3	J3.3	C3	12
pSTMos.11	ASSR	TALRP(LGE)HAL	YGELHFGPGTRLTVL	FM993922	D2	J2.1	C2	20
pSTMos.12	ASSPT	PT	YDERHFGPGTRLTVL	FM993923	--	J3.1	C3	12
pSTMos.13	ASSR	P(WGQG)D	GELHFGPGTRLTVL	FM993924	D3	J2.1	C2	14
pSTMos.14	ASS	G	SNNPLYFGGGTRLLVL	FM993925	--	J2.3	C2	10
pSTMos.17	ASSQD	M(G)ASA	AQLYFGAGSKLTVL	FM993926	D3	J3.2	C3	14
pSTMos.18	ASSQE	(GA)DH	NPLYFGGGTRLLVL	FM993927	D3	**J2.3**	**C3**	13
pSTMos.19	ASSQ	W	SNQAQHFGHGTRLAVL	FM993928	--	J1.5	C1	11
pSTMos.20	ASSPT	LR(G)IIPT	YEQYFGPGTKLTVV	FM993929	D1	**J2.7**	**C3**	16
pSTMos.21	ASS	RSR(WG)QD	SERYFGAGTRLTVT	FM993930	D3	J3.5	C3	14
pSTMos.22	ASSR	(WG)QD	SERYFGAGTRLTVT	FM993931	D3	J3.5	C3	12
pSTMos.23	ASSQ	APYG(TF)	SETQYFGPGTRLLVL	FM993932	*D2*	*J3.4*	C3	15
pSTMos.25	ASSPT	R(Q)GG	DPLYFGAGSKLTVL	FM993933	D1	**J2.2**	**C3**	13
pSSAR.01	ASSQ	SRR(D)VS	QTQYFGPGTRLLVL	FM993934	D1	J2.5	C2	14
pSSAR.02	ASSQG	HR(TA)K	NERLYFGNGTKLSVL	FM993935	D1	J1.4	C1	15
pSSAR.04	ASSQ	(AGGW)AL	SETQYFGPGTRLLVL	FM993936	D3	J3.4	C3	15
pSSAR.05	ASSK	LG(R)DILN	EQYFGPGTKLTVV	FM993937	D1	**J2.7**	**C3**	14
pSSAR.07	ASSQD	S(GTA)D	ERLYFGNGTKLSVL	FM993938	D1	J1.4	C1	14
pSSAR.08	ASS	(LG)	NERLYFGNGTKLSVL	FM993939	*D3*	*J1.4*	C1	10
pSSAR.10	ASSL	DI(R)PN	GELHFGPGTRLTVL	FM993940	D2	**J2.1**	**C3**	13
pSSAR.11	ASSP	K(RD)GY	NPLYFGGGTRLLVL	FM993941	D1	J2.3	C2	13
pSSAR.16	ASSQE	Q(Q)SRF	NNPLYFGGGTRLLVL	FM993942	D1	**J2.3**	**C1**	15
pSSAR.17	ASSQ	SRRD	SNQAQHFGHGTRLAIL	FM993943	--	J1.5	C1	14
pSSAR.19	ASSP	S(DFG)IG	NNPLYFGGGTRLLVL	FM993944	D2	J2.3	C2	15
pSSAR.23	ASS	LSTVD	SQSTQYFGAGTRLSVL	FM993945	--	J3.3	C3	14
pSSAR.24	ASSQD	RKQGG	NSPLQFGIGTRLTVT	FM993946	--	J1.6	C1	15
pSSAR.25	ASS	FFST(G)E	ETQYFGPGTRLLVL	FM993947	D3	J3.4	C3	13
pSSAR.28	ASSQ	(GQ)DRI	NPOLYFGGGTRLLVL	FM993948	D1	J2.3	C2	14
pSSAR.31	ASSP	DP(DSG)A	AQLYFGAGSKLTVL	FM993949	D1	J3.2	C3	14
pSSAR.32	ASSQD	IS(QR)A	TDPLYFGAGSKLTVL	FM993950	D1	**J2.2**	**C3**	15
pSMA.09	ASSP	(LWGG)D	NPLYFGGGTRLLVL	FM993951	D2	J2.3	C2	13
pSMA.10	ASSQD	(AG)F	NPLYFGGGTRLLVL	FM993952	D3	J2.3	C2	12
pSMA.41	ASSQ	KR(TAG)	ERHFGPGTRLTVL	FM993953	D1	J3.1	C3	12
pSMA.42	ASS	L(GQRG)G	YEQYFGPGTKLTVV	FM993954	D1	J2.7	C2	13
pSMA.46	ASSQD	I	ETQYFGPGTRLLVL	FM993955	**--**	J3.4	C2	10
pSMA.48	ASSQE	LT	YEQYFGPGTKLTVV	FM993956	**--**	**J2.7**	**C3**	11
pSMA.55	ASSR	(DL)C	NNPLYFGGGTRLLVL	FM993957	D2	**J2.3**	**C3**	12
pSMA.59	ASSS	V(S)D	GELHFGPGTRLTVL	FM993958	D1	J2.1	C2	11
pSMA.60	ASSP	AV(G)SD	NPLYFGGGTRLLVL	FM993959	D1	**J2.3**	**C3**	13
pSMA.62	ASSP	Q(TAG)E	DPLYFGAGSKLTVL	FM993960	D1	J2.2	C2	13
pSMA.65	ASSK	GR(TAG)P	SNNPLYFGGGTRLLVL	FM993961	D1	**J2.3**	**C3**	16
pSMA.66	ASSS	DR(GW)S	QTQYFGPGTRLLVL	FM993962	D3	J2.5	C2	13
pSMA.67	ASSK	TDF	YEQYFGPGTKLTVV	FM993963	--	J2.7	C2	11
pSMA.68	ASS	W(T)LNA	AQLYFGAGSKLTVL	FM993964	D1	J3.2	C2	12
pSMA.70	ASSR	E(GTG)L	YEQYFGPGTKLTVV	FM993965	D1	**J2.7**	**C3**	13
pSMA.71	ASSP	(GP)T	NTEVFFGKGTRLTVV	FM993966	D1	J1.1	C1	12
pSMA.73	ASSP	FL(DS)V	DERHFGPGTRLTVL	FM993967	D1	J3.1	C3	13
pSMA.74	ASS	R(H)QNI	TDTQYFGPGTRLSVL	FM993968	D1	J2.4	C2	13
pSMA.76	ASSP	(S)	SNNPLYFGGGTRLLVL	FM993969	D3	J2.3	C2	11
pSTA.01	ASSR	Y(SE)GD	EYHFGPGTKLTVV	FM993970	D1	J1.2	C3	12
pSTA.02	ASSQD	LV(GTA)R	YEYHFGPGTKLTVV	FM993971	D1	J1.2	C3	15
pSTA.03	ASS	FFST(G)E	ETQYFGPGTRLLVL	FM993972	D3	J3.4	C3	13
pSTA.04	ASS	DS(W)DVQS	TQYFGPGTRLLVL	FM993973	D3	J3.4	C3	13
pSTA.06	ASSQD	(R)D	YEYHFGPGTKLTVV	FM993974	D1	J1.2	C3	11
pSTA.08	ASSP	S(R)D	TEVFFGKGTRLTVV	FM993975	D1	J1.1	C3	11
pSTA.09	ASS	(LR)	ETQYFGPGTRLLVL	FM993976	*D2*	*J3.4*	C3	9
pSTA.11	ASSP	(GQR)PP	DTQYFGPGTRLSVL	FM993977	D1	J2.4	C2	13
pSTA.12	ASS	LS(GTRG)D	TQTQYFGPGTRLLVL	FM993978	D1	J2.5	C2	15
pSTA.13	ASSR	K(RG)H	SETQYFGPGTRLLVL	FM993979	D1	J3.4	C3	13
pSTA.15	ASS	IEKA	NPLYFGGGTRLLVL	FM993980	--	J2.3	C2	11
pSTA.24	ASSK	DL(AGG)VS	SETQYFGPGTRLLVL	FM993981	D3	J3.4	C2	16
pSTA.25	ASSL	ER(QR)	DERHFGPGTRLTVL	FM993982	D1	J3.1	C3	12
pSTA.26	ASSP	R(S)DK	GELHFGPGTRLTVL	FM993983	D1	**J2.1**	**C1**	12
pSTA.29	ASSP	RQ(T)GPFG	EYHFGPGTKLTVV	FM993984	D1	J1.2	C1	14

Three nucleotide differences at the N-terminus allow to distinguish the three TRBC gene isotypes: *TRBC1 *differs with respect to *TRBC2 *and *TRBC3 *genes for two nucleotide substitutions in the third and fourth codons; *TRBC3 *(as well as *TRBC1 *gene) is distinguishable from *TRBC2 *because of a silent nucleotide substitution at the third position of the first codon [12]. On the basis of these criteria, the N-terminus of the TRBC portions within the cDNA sequences was analyzed and a significant group of cDNAs with the *TRBC3 *gene (35/72 = 48.6%) identified. Moreover, 25 clones retain the *TRBC2 *(34.7%) and 12 clones are with the *TRBC1 *(16.6%) gene (Table 1).

More complex is the determination and the contribution of the genes involved in the CDR3 formation. The CDR3 β region is defined as a stretch of nucleotides running after the codon encoding the cystein in position 104 of the *TRBV *gene to the codon before that which encodes the phenylalanine of the FGXG motif of the *TRBJ *gene http://imgt.cines.fr/[17]. The corresponding amino acid sequence of the CDR3 loop deduced from the nucleotide sequences reveals that it is heterogeneous for amino acid composition (Table 1). The mean length of the CDR3 loop was approximately the same in spleen (mean 12.3 aa, range 10-16 aa) and adult thymus (mean 12.6 aa, range 9-16 aa), but larger in blood (mean 13.9 aa, range 10-15 aa) and young thymus (mean 13.7 aa, range 10-20 aa). For comparison, human peripheral blood CDR3β loop is about 12.7 residue long [18] and mouse is 11.9 residue long [19]. A similar CDR3 length and size range was reported in thymus and peripheral blood lymphocytes of piglets (mean 13.1 aa, range 10-17 aa) [20].

**Figure 2:**
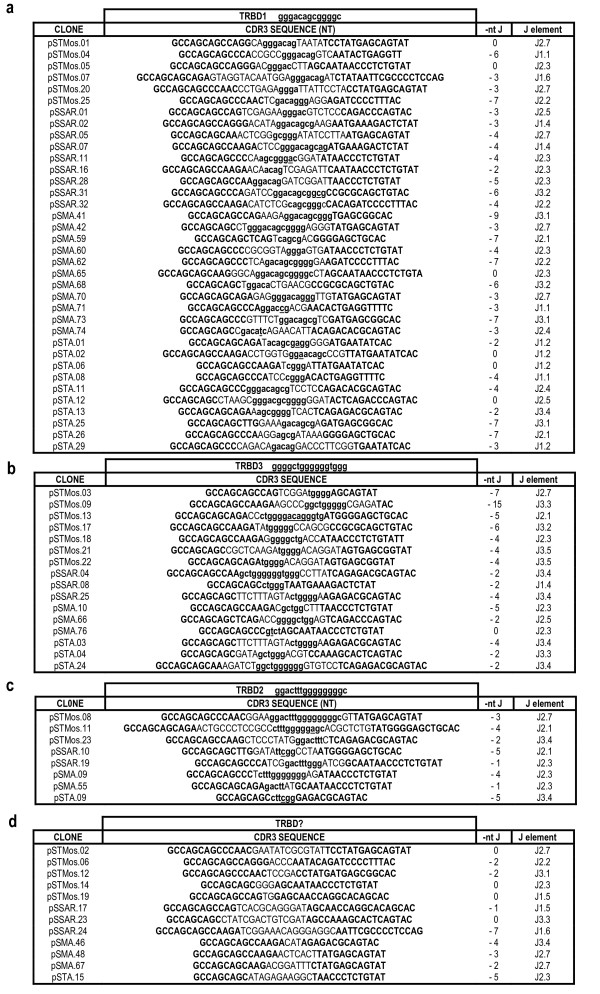
**CDR3 nucleotide sequences retrieved from the cDNA clones**. Sequences are shown from the codon after the *cys-94 *of the *TRBV *gene to the codon before the *phe-104 *of the *TRBJ *gene and grouped on the basis of the *TRBD1 *(a), *TRBD3 *(b), *TRBD2 *(c) or no *TRBD *usage (d). Nucleotides that are conserved in the 3' end of the V portion are considered of TRBV genomic origin and indicated in bold upper cases. Residues belonging to the different *TRBJ *genes, on the right, are indicated also in bold upper case at the 3' end of each sequence. The germline sequences of *TRBD1*, *TRBD3 *and *TRBD2 *gene are indicated at the top of each figure. The sequences considered to present recognizable *TRBD *genes (see text) are indicated in lower cases and nucleotide substitutions or insertions are underlined. Nucleotides that cannot be attributed to any coding elements (N-nucleotides) are indicated in capital letters on the left and on the right sides of the TRBD regions. Numbers in the right column indicate the level of 5'- *TRBJ *nucleotide trimming.

For a close inspection of the CDR3 s, the nucleotide sequences have been excised from each cDNA sequence and analyzed in detail. In the absence of the TRBV germline sequences, the deletions at the 3'-end of the *TRBV *and the nucleotide addition at the V-D junctions cannot be accurately estimated. However, the comparison of the 72 V-D-J junctions after the ASS motif allowed the determination of the probable 3'-end of the *TRBV *gene that has not been trimmed by exonuclease during rearrangement in a significant proportion of sequences (Table 1). By the comparison of the *TRBD *genomic sequences, the nucleotides located in the CDR3 regions were considered to belong to a *TRBD *gene if they constituted a stretch of at least four consecutive residues corresponding to the *TRBD1*, *TRBD3 *or *TRBD2 *germline sequences. In this way the 72 sequences were grouped according to the *TRBD1 *(fig. 2a, 36 sequences), *TRBD3 *(fig. 2b, 16 sequences) or *TRBD2 *(fig. 2c, 8 sequences) gene usage. 12 sequences with no recognizable *TRBD *genes were grouped separately (fig. 2d). These last sequences could be interpreted as direct V-J junctions. However, it is also possible that nucleotide trimming masked the initial participation of D gene during the rearrangement. In the other cases the degree of germline nucleotide trimming in the 3'-V and 5'-J as well as the 5' and 3' D region is similar in all groups (fig. 2). Nucleotides that could not be attributed to any template sequence are considered N-elements. The mean length for N-D-N addition, including D region, is 15 nt (range 6-23 bases) for the first group (fig. 2a), 13.8 nt (range 4-22 bases) for the second group (fig. 2b) and 16 nt (range 6-33 bases) for the group with *TRBD2 *participation (fig. 2c). The mean of N addition in the clones without *TRBD *sequence (fig. 2d) is 8.3 nt (range 2-16 bases). Particular features of the CDR3 region of the clones are the presence within the D region of nucleotide substitutions as well as the presence of insertion (psTMos 13 in fig. 2b) and deletion (psTA12 in fig. 2a) with respect to the germline sequences. Although the numbers are too low to be statistically relevant, a trend towards longer CDR3 length in TRBD2 (mean 42.3 bp, range 27-60) with respect to TRBD1 (mean 40.3 bp range 33-54) and TRBD3 (mean 38.5 bp, range 30-48), or with no apparent TRBD (mean 36.2 bp, range 30-42) transcripts was evident.

These data together suggest that all three *TRB *D-J-C clusters are used to generate in sheep functional TR β-chain with no specific influence of any clusters.

### Analysis of the D-J-C rearrangements

Since the genomic organization of the 3' region of the sheep *TRB *locus is known (fig. 1) [12], the formal interpretation of the D-J-C arrangements is possible. The intra-cluster rearrangements represent a consistent portion of the repertoire (41.6%), with 10 *TRBD1*-*TRBJ1*, 9 *TRBD3*-*TRBJ3 *and 6 *TRBD2*-*TRBJ2 *rearrangements (Table 1). A similar number of rearrangements (53.3%) can be interpreted by direct 5'- to- 3' joining across the clusters (inter-cluster rearrangements) with 20 *TRBD1*-*TRBJ2*, 6 *TRBD1*-*TRBJ3 *and 6 *TRBD3*-*TRBJ2 *rearrangements (Table 1). Interestingly, we also observed two *TRBD2*-*TRBJ3 *(psTMOs23 and psTA09, italics in Table 1) and one *TRBD3*-*TRBJ1 *(psSAR08, italics in Table 1) joining. Since the D- J-C cluster 2 is located downstream D- J-C cluster 3 as well as D- J-C cluster 3 is downstream D- J-C cluster 1 within the *TRB *locus, both these junctions may only be explained by chromosomal inversion, or with more probability, by trans-rearrangement occurring during *TRB *locus recombination.

A systematic analysis of the constant region of the transcripts also revealed that multiple splice variants are present. In fact, the canonical splicing is present in 49/72 (68%) clones with 10 *TRBJ1*-*TRBC1*, 17 *TRBJ3*-*TRBC3 *and 22 *TRBJ2*-*TRBC2 *transcripts (Table 1). A group of 7 clones (4 *TRBJ1*-*TRBC3 *and 3 *TRBJ3*-*TRBC2*) comes from an alternative or cis-splicing mechanism (9.7%). Finally, it is noteworthy that 16 clones (22.2%, bold in Table 1) with *TRBJ2 *genes showed *TRBC3 *or *TRBC1 *instead of the expected *TRBC2 *gene. Since *TRBC3 *as well as *TRBC1 *genes are located upstream TRBJ2 cluster in the germline DNA, *TRBJ2 *joined to *TRBC1 *or *TRBC3 *sequences cannot be a cis-spliced product of a single precursor RNA. Consequently, they must be the product of a trans-splicing between a transcript with *TRBJ2*-*TRBC2 *genes and a transcript containing *TRBC1 *or *TRBC3 *genes.

We excluded that all these non canonical sequences may be the result of PCR artifacts since the crossover points have not as expected a random distribution, but they always lie at the D-J or/and J-C junction, giving rise to products of the appropriate length and sequence.

The presence of splice variants may suggest the involvement of the *TRBC *gene in generating the TR β-chain functional diversity.

### Structure of the TRBC region

To complete the analysis of the TRBC domain in the cDNA clones, the whole constant portion of the transcripts was retrieved from the sequences and aligned according to the three *TRBC *isotypes for each animal in the different tissues.

**Figure 3:**
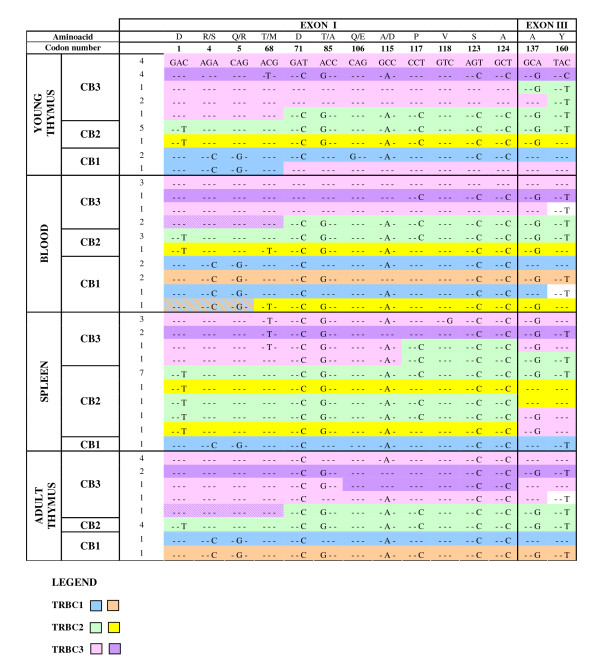
**The nucleotide sequences of the *TRBC *isotypes derived from the cDNA clones**. Only the 14 variable nucleotide codons (12 in the first and two in the third exons numbered from the first position of the constant region in the cDNA) are depicted. The amino acids specified by the corresponding codons and those due to the nucleotide substitutions are given at the top of each codon, using the single letter code. The sequences are organized with respect to the one allelic TRBC3 sequence isolated from *Moscia Leccese *breed young thymus. Identities of the other allelic form of the same gene or of the other *TRBC *isotypes in the other tissues with respect to the reference sequence are indicated by dashes, while nucleotide substitutions are shown. The number on the left indicates the clones with the corresponding sequences. All the allelic forms of the TRBC isotypes are identified by a color. Color changes indicate recombinational diversification or alternative splicing.

The comparison of the 72 cDNAs showed the presence of different sequences that can be identified for the nucleotide variability in 14 different positions, 12 located in the first and two in the third exon, resulting in six amino acid substitutions all grouped in the first exon, and as a consequence, in the extracellular domain of the chain (fig. 3). By means of these variations, we observed a number of different sequences in excess. For example, five different groups of sequences were assigned to the *TRBC3 *gene in the young thymus of the *Moscia Leccese *breed individual. This number is certainly higher than the expected two allelic forms, at the most, of the gene. In order to understand the origin of the additional sequences, we have isolated by PCR the allelic variants of all three *TRBC *genes from the young thymus genomic DNA of the *Moscia Leccese *individual, used as a reference model with respect to the others. The specificity of the PCR reactions was achieved by using a reverse primer which binds to either *TRBC1 *and *TRBC3 *(B40) or *TRBC2 *3'-UTR (B42) sequences, and completely TRBC specific forward primers complementary to a specific region upstream the *TRBC1 *(CC1), *TRBC3 *(CC3) and *TRBC2 *(CC2) coding regions (see Methods). The three different PCR products were sequenced, and in every case, two allelic forms for each *TRBC *gene were obtained (data not shown). The comparison of the genomic with the corresponding sequences within the young thymus cDNAs allows us to establish that the first two more abundant groups of TRBC3 sequences represent the two allelic forms of the *TRBC3 *genes (pink and lilac in fig. 3), while alternative splicing of the third exon and DNA recombinational diversification process with the *TRBC2 *gene can have generated the other three groups of TRBC3 sequences (mixed color in fig. 3). Moreover, the two groups of TRBC2 cDNA sequences (green and yellow in fig. 3) perfectly matched with the two allelic forms (data not shown). Only one allelic form was recovered for the *TRBC1 *gene (italics in fig. 3), while the other TRBC1 sequence can have been generated by a mechanism of DNA recombinational diversification with the allele of *TRBC3 *gene (mixed color).

After deducing the allelic variants of the three constant genes in the other tissues, alternative splicing and recombinational diversification can explain the excess of the sequences also in those cases.

## Discussion

To validate the real participation of the third additional D-J-C cluster and compare its usage with respect to the others in the formation of the TR β-chain repertoire, we analyzed transcripts of 72 unique D-J-C rearrangements recovered from four different tissues of four different animals, belonging to three different ovine breeds. Although the analyzed sequences lacked the *TRBV *genes, the presence of the CDR3 β region, the *TRBJ *gene as well as most of the *TRBC *gene sequence was sufficient to permit a comprehensive analysis of the expressed TR β chain. Data presented here show that the mechanisms for generating diversity in sheep β chain polypeptides appear to adhere to the paradigms established through the study of humans and rodents. However, the diversity is enhanced by somatic rearrangement of 3 *TRBD *and 17 *TRBJ *genes that, by virtue of the expected recombination imprecision and N-region addition, maximizes diversity in the CDR3 region, thus expanding the potential repertoire of antigen specificities (Table 1). In spite of the presence of a longer coding nucleotide sequence in *TRBD *genes if compared with the human and mouse counterpart [12], the overall size of the CDR3 region is conserved in all tissues among the different mammalian species (Table 1). This conservation was archived by a greater deletion at the 5'end of *TRBJ *genes and a concomitant increase in N-nucleotide addition at the V-D-J junction during rearrangement (fig. 2). This suggests that the length of CDR3 in TR β chain is essential for T-cell function.

While there is not a specific influence of any cluster in the formation of the sheep β-chain in the different tissues, a dissimilar usage of the genes can be identified and it could depend on the sheep *TRB *genomic organization. Consistent with a promoter-enhancer facilitated recombination model, in human and mouse, assembly of the DJβ1 cassette is dependent on the interaction of the enhancer with the PDβ1 promoter positioned immediately 5' of the *TRBD1 *gene. Assembly of DJβ2 proceeds independent from that of DJβ1, albeit with less efficiency. Also in this case, an undefined PDβ2 region continues to associate with the enhancer [21]. Our analyses suggest that also in sheep the mechanisms selectively alter D usage, so that the "privileged" *TRBD1 *gene can account for the 60% of the total clones with respect to 26.6% of *TRBD3 *and 13.3% of *TRBD2*. This may reside in the greater efficiency of the PDβ1 promoter activity with respect to the PDβ3 or PDβ2. A striking conservation of the PDβ1 and PDβ2 (as well as PDβ3) regions among sheep, human and mouse [12] can support this observation, whereas the activity of the two similar PDβ3 and PDβ2 promoters could be correlated with their position from 5' to 3' within the locus.

The prominent utilization of the members of the *TRBJ2 *with respect to the *TRBJ3 *and *TRBJ1 *sets, as deducted from our cDNA collection, results from inter cluster or trans-rearrangements. It is possible that the preferential usage of the *TRBJ2 *set could depend on the number of genes that lie in the genomic region, if multiple Jβ 12-RSs are important for increasing the local concentration of the RAG proteins that first bind a 12-RS and then capture a 23-RS to form a synaptic complex [22]. In this regard, it is notable that the six sheep *TRBJ1 *genes lie in about 2.1 Kb, the five *TRBJ3 *genes in about 900 bp, while the seven *TRBJ2 *genes are grouped in about 1 Kb. Recently, Franchini et al. [23] have demonstrated, by means of an *in vitro *RAG1/2 mediated DNA coupled cleavage assay using various pair-wise RS combinations, that in mouse, the coupled cleavage of Dβ1-Jβ1 and Dβ2-Jβ2 substrates are similar and are both weak if compared to Dβ1-Jβ2 substrates, suggesting that Jβ2 RSs are better partners than Jβ1 RSs. In the same way, in sheep there could be the presence of a hierarchy efficiency of coupled cleavage with the Dβ1-Jβ2 > Dβ1-Jβ3 > Dβ1-Jβ1.

As the increment of the number of *TRBD *and *TRBJ *genes produces larger variation in TR β chain, particularly in CDR3 region as expected, similarly, the presence of an additional *TRBC *gene seems to affect the variety of the β chain repertoire. In fact careful analysis of the cDNA constant regions obtained from the different animals showed a level of unexpected variability in the first exon of the *TRBC *genes (fig. 3) if compared with that established in the genomic sequence [12]. By using the single nucleotide variations present in the first and third exon of the *TRBC *genes as hallmarks, we demonstrated that alternative splicing concerning the first and/or the third exon and/or somatic recombinatiorial processes are involved in the diversification of the constant region of the sheep β-chain. The alternative splicing can occur either in cis or in trans. The presence of a cis-splicing mechanism comes from the analysis of six clones with *TRBJ1*-*TRBC3 *and *TRBJ3*-*TRBC2 *arrangement, while the presence of a trans-splicing process derives from the analysis of 16 clones with *TRBJ2 *spliced to *TRBC3 *or *TRBC1 *instead of the expected *TRBC2 *gene (Table 1). *TRBJ2 *to *TRBC1 *or *TRBC3 *splicing could be possible only when TRBV-TRBD-TRBJ transcripts are spliced with a transcript of the other allele. As a consequence, trans-splicing of two RNA separate precursors is the only logical possibility. The involvement of interallelic trans-splicing has already been documented in IgH chains [24]. Beyond this case the presence of interallelic trans-splicing in vertebrates is problematical to demonstrate. It has been documented to be an essential process for the expression of the *lola *Drosophila gene. *Lola *encodes 20 protein isoforms belonging to a family of BTB zinc-finger transcriptional factor [25]. Genetic tests have demonstrated that some isoforms were generating thought intrallelic trans-splicing [26]. No particular sequences for trans-splicing have been identified around the exon-intron boundary in the *lola *gene; therefore, the basic mechanism of trans-splicing is likely to be shared with those of cis-splicing and occur co-transcriptionally where nascent pre mRNA are produced in close proximity, as is the case for cis-splicing [26]. It is possible that also in sheep *TRB *locus, the cis and trans-splicing shared the same mechanism.

Investigation of the constant domain of the sheep cDNAs led us to deduce that a minimal set of sequences are also generated by a somatic recombinatorial process (fig. 3). Somatic recombinatorial diversification occurs in vertebrates, yeast and plants [27-29], and such a modification of germline sequences can generate individuals with different starting gene repertoires in different tissues.

The precise effect and significance of the variability in the constant region of TR β-chain remain to be determined. It might create diversity in the T cell function. The extracellular domain of the TRBC molecule consists of well-defined regions [15]. The pattern of amino acid replacements in the sheep cDNA was located, beyond the N- terminus, one both in the TRBC E β-strand and in the DE loop and two in the FG loop. This last is TR β-chain specific loop in all mammalian species and contains 12 residues that are conserved between the two TRBC isotypes in human and mouse. In sheep sequences, the FG loop is one amino acid longer and underwent replacement among the three *TRBC *genes. So the Gln in position 106 in the first half part of the loop can be replaced by Glu; while the Asp in position 115 of the second part of the loop can be substituted by Ala (fig. 3). Three-dimensional structures of the TR [30] have shown that the FG loop of the TR β chain exists as an elongated, rigid element forming a sidewall of a cavity created by the asymmetric disposition of Cα and Cβ domains that receive the ε subunit of the CD3 complex [31]. Therefore a primary function of the Cβ FG loop in the thymus is to facilitate negative selection, while following maturation, αβ T cells are dependent on the Cβ FG loop to their activation [32]. Our hypothesis is that amino acid replacement in the FG loop of the sheep *TRBC *genes can be modified by the sensitivity of αβ T cell for cognate peptide recognition, and this can be correlated with the function of the αβ T cell in sheep.

## Conclusions

All together our results show that in sheep the presence of an additional D-J-C cluster enhances the β-chain repertoire. These findings, together with the evidence of the expansion of gene repertoires for other TR loci in ruminants [33-35], suggest that strong evolutionary pressures have driven a generic enlargement of TR gene numbers, thus generating a greater potential TR diversity in this lineage.

## Methods

### Animals (source of tissue)

Thymus, spleen and blood were obtained from animals of three different autochthonous breeds. One thymus was collected from one neonatal *Moscia Leccese *sheep; spleen and the other thymus from one adult *Gentile di Puglia*; and blood from one young *Sarda Ionica *sheep. All animals were conventionally reared outbred sheep and were healthy at the time of sample collection. All animal manipulations were carried out with the approval of the Bari Animal Ethics Commitee and in compliance with Institutional Animal Care and Use Comittee (IACUC) requirements.

### RT-PCR

The different organs were removed from the animals, immediately frozen in liquid nitrogen and stored at -70°C until preparation of RNA. In the case of blood, RNA was prepared before freezing.

Total RNA was extracted from tissues under the protocol approved by the manufacturer (Trizol reagent, Invitrogen). First-strand cDNA synthesis was performed by reverse transcription of 5 μgr of total RNA primed with 2,5 μl of oligodT (0,5 μg/μl) using 2 μl dNTP (10 mM), 2 μl DTT (100 mM) and 1 μl PowerScript™ReverseTrancriptase (Clontech) in the recommended buffer in a total of 20 μl. The genes of interest were amplified from 10% of cDNA preparations using a sense V primer (VB3; 5'-TATCTCTGTGCCAGCAGC-3') complementary to a conserved region in the 3'-end of sheep *TRBV *genes [16] and an antisense CB3 primer (5'-CACCAGGGCGCTGACCAG-3'; AM420900; 8,222-8,239 positions) (5'-CACCAGGGCGCTGACCAG-3'; AM420900; 17,442-17,459 positions) (5'-CACCAGGGCGCTGACCAG-3'; AM420900; 26,702-26,719 positions) located in the third exon of the sheep *TRBC *genes. All the PCR were performed in a 50 μl volume with 5 μl 10× buffer, 2 μl MgCl2 (50 mM), 1 μl dNTP (10 mM), 0,5 μl of Taq Platinuum 5 U/μl (Invitrogen) and 2 μl of the sense and antisense primers (10 mM). After 2 min of initial denaturation at 94°C, the samples were subjected to 35 cycles of amplification (30 s at 94°C, 30 s at 58°C, 30 s at 72°C). The final cycle was extended for 10 min at 72°C. Amplified cDNA fragments were purified by using the PureLink PCR Purification Kit (Invitrogen-Life Technologies), ligated into StrataClone PCR Cloning Vector and transformed into StrataClone Competent Cells (Stratagene).

### DNA amplification

Genomic DNA was isolated from the young *Moscia Leccese *thymus by standard techniques. For the DNA amplifications, 50-200 μgr of thymus DNA was used with the TaKaRa LA Taq in 50 μl reactions, according to the recommendations (TAKARA BIO INC.). The cycling conditions were as follows: 94°C for 1 min; 35 cycles of 30 s denaturation at 95°C, 1 min annealing at 58°C, 2 min polymerization at 68°C; and 68°C for 10 min. The primer combinations used were CC1 and B40 for the *TRBC1 *gene, CC3 and B40 for the *TRBC3 *gene and CC2 and B42 for the *TRBC2 *gene. The CC1 (5'-CTGTGGCCCCTTTCCTTGTT-3'; AM420900, 6,805-6,824 positions), CC3 (5'-ACACACACAGCCCCTACCA-3', AM420900, 16,324-16,342 positions) and CC2 (5'-AGAGATGGGTTGTCGTAGG-3', AM420900, 25,117-25,136 positions) are designed on the 5'- end specific of the *TRBC1*, *TRBC3 *and *TRBC2 *genes respectively. B40 (5'-TCAGGGCAGTAACAGGCT-3'; AM420900, 8587-8569 positions) (5'-TCAGGGCAGTAACAGGCT-3'; AM420900; 17832-17815 positions) is complementary to the 3'UTR of *TRBC1 *as well as *TRBC3 *genes, while B42 (5'-ATGACTCGGGACGCACTT-3', AM420900, 27,040-27,057 positions) is complementary to the 3'UTR of the *TRBC2 *genes. Amplified DNA fragments were purified by PureLink PCR Purification Kit (Invitrogen-Life Technologies) and used directly for DNA sequencing.

### Determination of CDR3 length and sequence analyses

The CDR3 size was calculated by the number of amino acids between the amino acid after the conserved 2^nd ^cysteine in the V gene (pos.104), and the amino acid before the phenylalanina of the FGXG motif in the J gene http://imgt.cines.fr/[17]. This method gives the CDR3 length with three amino acids more than that done in Kabat et al [36].

Nucleotide sequences were determined by a commercial service. DNA sequence data were processed and analyzed using the blasta program http://www.ncbi.nlm.nih.gov/BLAST, Clustal W http://www.ebi.ac.uk/clustalw/index.html[37] and IMGT database http://imgt.cines.fr/) [17].

## Abbreviations

TR: T cell receptor; TRB: T cell receptor beta; TRBV: T cell receptor beta variable gene; TRBJ: T cell receptor beta joining gene; TRBD: T cell receptor beta diversity gene; TRBC: T cell receptor beta constant gene.

## Authors' contributions

SDT carried out the molecular studies and analyzed data. RA participated in the design of the study and helped to draft the manuscript. SC revised the manuscript critically. SM conceived of the study, participated in its design and coordination and wrote the manuscript. All authors have read and approved the final manuscript.
